# Allogeneic and autogenous transplantations of MSCs in treatment of the physeal bone bridge in rabbits

**DOI:** 10.1186/1472-6750-8-70

**Published:** 2008-09-12

**Authors:** Ladislav Planka, Petr Gal, Helga Kecova, Jiri Klima, Jana Hlucilova, Eva Filova, Evzen Amler, Petr Krupa, Leos Kren, Robert Srnec, Lucie Urbanova, Jana Lorenzova, Alois Necas

**Affiliations:** 1 Department of Pediatric Surgery, Orthopaedics and Traumatology, the Faculty Hospital Brno, Jihlavska 20, Brno, Czech Republic; 2 Department of Surgery and Orthopaedics, Small Animal Clinic, Faculty of Veterinary Medicine, University of Veterinary and Pharmaceutical Sciences Brno, Palackeho 1-3, Brno, Czech Republic; 3 Institute of Animal Physiology and Genetics of the Academy of Sciences of the Czech Republic, Rumburska 86, Libechov, Czech Republic; 4 Institute of Experimental Medicine of the Academy of Sciences of the Czech Republic, Videnska 1083, Prague, Czech Republic; 5 Department of Medical Imaging, St. Anne's University Hospital, Masaryk University, Pekarska 53, Brno, Czech Republic; 6 Department of Pathology, the Faculty Hospital Brno, Jihlavska 20, Brno, Czech Republic

## Abstract

**Background:**

The aim of this experimental study on New Zealand's white rabbits was to find differences in the results of treating the distal physeal femoral defect by the transplantation of autologous or allogeneic mesenchymal stem cells (MSCs). After the excision of a created bone bridge in the distal physis of the right femur, modified composite scaffold with MSCs was transplanted into the defect. In animal Group A (n = 11) autogenous MSCs were implanted; in animal Group B (n = 15) allogeneic MSCs were implanted. An iatrogenic physeal defect of the left femur of each animal not treated by MSCs transplantation served as control. The rabbits were euthanized four months after the transplantation. The treatment results were evaluated morphometrically (femoral length and valgus deformity measurement) and histologically (character and quality of the new cartilage).

**Results:**

Four months after the transplantation, the right femurs of the animals in Group A were on average longer by 0.50 ± 0.04 cm (p = 0.018) than their left femurs, the right femurs of rabbits in Group B were on average longer by 0.43 ± 0.01 cm (p = 0.028) than their left femurs.

4 months after the therapeutic transplantation of MSCs valgus deformity of the distal part of the right femur of animals in Group A was significantly lower (by 4.45 ± 1.86°) than that of their left femur (p = 0.028), in Group B as well (by 3.66 ± 0.95° than that of their left femur p = 0.001). However, no significant difference was found between rabbits with transplanted autogenous MSCs (Group A) and rabbits with transplanted allogeneic MSCs (Group B) either in the femur length (p = 0.495), or in its valgus deformity (p = 0.1597). After the MSCs transplantation the presence of a newly formed hyaline cartilage was demonstrated histologically in all the animals (both groups). The ability of transplanted MSCs to survive in the damaged physis was demonstrated in vivo by magnetic resonance, in vitro by Perls reaction and immunofluorescence.

**Conclusion:**

The transplantation of both autogenous and allogeneic MSCs into a defect of the growth plate appears as an effective method of surgical treatment of physeal cartilage injury. However, the Findings point to the conclusion that there is no clear difference in the final effect of the transplantation procedure used.

## Background

By impairment of enchondral ossification and normal chondrogenesis in the area of growth cartilage of the long bones of the extremities, a formation of the bone bridge may occur with a subsequent disturbance of bone growth [1,2]. For a number of years, rooted methods of treatment of the physeal plate closure due to its trauma have been passed on [3-5]. However, considering their exigence of time and finances and the seriousness of possible complications, different procedures have been searched for in experiments, that would allow correcting deformities (or even to prevent the development of such deformities) of the long bones of the extremities with an injured physis less invasively and with a better clinical effect and lower percentage of complications. Some departments have started to focus their attention on transplantations of tissues and cellular colonies into the defect with the aim to restore physeal morphology and function. In the past six years we have pursued experiments with the transplantation of chondrocytes and mesenchymal stem cells (MSCs) into the damaged growth plate with the purpose to restore its function (transplantation of autologous chondrocytes into an iatrogenically injured growth plate of the distal femur of the pig, prevention of formation of a bone bridge by transplantation of allogeneic MSCs into an iatrogenically created defect, therapy of a formed bone bridge by its excision and transplantation of allogeneic stem cells into the created defect) [6-9]. Yet, in the area of cellular transplantation a number of questions remain unanswered before their possible clinical use in humans can happen. One of these questions is also the aspects of autogenous vs. allogeneic transplantations of mesenchymal stem cells into the injured physeal growth zone of the extremity bones.

The study of possible repair of the growth cartilage tissue has notably benefited and advanced thanks to works addressing the transplantation of autogenous chondrocytes into the growth cartilage defect [10-14]. In practice, transplantation of autogenous chondrocytes represents the collection of the cartilaginous tissue and subsequently the cultivation of a chondrocyte graft that may be used for the autogenous transplantation into a growth cartilage defect after the lapse of approximately 3 weeks [15]. This time lag between the autograft sampling and on possibility to implant cultivated cells might limit the autotransplantation in actual clinical practice. Therefore, allograft transplantation appears suitable in this regard, offering, provided the availability of a tissue bank, the possibility of immediate cell transplantation into the damaged target tissue.

The aim of this experimental study focusing on the surgical treatment of an iatrogenically created bone bridge in the distal physis of the femur in rabbits in form of its excision and subsequent transplantation of MSCs into the physeal defect, was to find whether there would be differences between the results of treatment by transplantation of autologous mesenchymal stem cells and by transplantation of allogeneic MSCs, always in the same gel scaffold. We evaluated the results of treatment partly on the basis of measurements of the length and valgus deformity of the femurs (morphometric variables of bone growth) and partly with regard to the quality of the newly formed cartilaginous tissue at the site of the original physeal defect (histological examination).

The proof that the newly formed chondrocytes at the site of the original physeal defect originated from the transplanted mesenchymal stem cells was established partly in vivo by demonstrating cells labelled with ferrous nanoparticles in the magnetic resonance examination and partly in vitro using Pearls reaction and immunofluorescence.

## Methods

The New Zealand white rabbit from a certified breeding was chosen as the experimental animal. The experiment included 26 healthy animals (12 males and 14 females) at the age of 5 weeks at the bone marrow collection (at the beginning of the experiment). The group of animals was homogenous as regards weight (2.32 ± 0.14 kg). The rabbits were divided into two groups: Group A (therapeutic autogenous transplantation of MSCs into the growth cartilage defect – 13 animals); and Group B (therapeutic allogeneic transplantation of MSCs into the growth cartilage defect – 13 animals). However, due to difficulties with MSC cultivation (the autogenous graft did not reach the needed number of cells on the day of transplantation) two experimental animals from Group A had to be transferred to Group B. Thus, the final number of animals evaluated in Group A was 11 and the final number of animals in Group B was 15.

### Preparation of the stem cells and the scaffold

Twenty one days before the transplantation of MSCs bone marrow blood was collected in general anaesthesia from the iliac wing on both sides. Using a punction needle (20 G/40 mm) an average of 2 ml blood was aspired from *tuber coxae alae osis illi *in to the 5 mL syringe with 2 mL Dulbecco's Phosphate Buffered Saline (PBS) with 2% Fetal Bovine Serum (FBS, StemCell Technologies) and 5 IU heparin/mL connected with a hypodermic needle (20 G/40 mm). Under sterile conditions, the bone marrow blood (about 4 mL) was deposited over 3 mL of Ficoll-Paque PLUS (StemCell Technologies). After centrifugation at 400 g for 30 min at room temperature, the dense gradient separated erythrocytes and granulocytes as a pellet in a bottom part of the tube; mononuclear cells were situated in an opalescent layer between Ficoll and blood plasma. This layer was taken out, washed in a culture medium (see below) and used for propagation under in vitro conditions. The average amount of mononuclear cells from each isolation was 20 × 10^6 ^cells. Cell number and viability was analyzed on Vi-CELL (Series Cell Viability Analyzers) and about 90% of viable cells were detected.

Cells were seeded in 75-cm2 tissue culture plastic flasks at a density of approximately 5 × 10^5 ^cells/cm2 and cultured at 37°C in humidified atmosphere with 5% of CO2. The culture medium was α-MEM medium (Gibco) supplemented with 10% FBS (Sigma Aldrich) and gentamycin (50 mg/mL, Sigma Aldrich). After 24 h of culture, the non-adherent cells were removed and during the subsequent culture (3 weeks) the medium was exchanged every third day. The first colonies of mesenchymal stem cells appeared after 4 to 5 days of culture and the 80% of confluence was achieved after 10 days of culture. Cells were passaged with trypsin 0.5% trypsin-EDTA solution (Sigma Aldrich) for 5 min at 37°C and replated in 150 cm2 at a density of 5000–6000 cells/cm^2^.

During the last three days of culture, cells were labeled with nano-particles of iron oxide (Resovist, 0.5 mmol Fe/mL, Schering). Resovist was added in concentration 1 μL/mL culture medium. For labeling with a fluorescent dye CM-DiI (Molecular Probes) in concentration 5 μg/2,5 mL PBS, cells were harvested on day of transplantation, incubated for 5 min at 37°C and for 15 min at 4°C. At the end of labeling cells were thoroughly washed in PBS. To induce chondrogenic differentiation [15], the labeled cells were given in the differentiation medium composed of α-MEM supplemented with 100 ng/mL recombinant human TGF β1 (R&D Systems), 100 nM dexamethasone (Medochemie), 50 μg/ml L-ascorbic acid 2-phosphate (Sigma Aldrich), 1% insulin-transferrin-selenium A (Gibco) for 30 min. Subsequently, the cells were centrifuged at 700 g for 5 min, and cell pellets were prepared for their deposition in a scaffold.

The scaffolds were prepared in a 96 – well plate at about 4°C by mixing 20,75 μL of sodium hyaluronate (10 mg/mL, 1500 kDa, kindly provided by CPN, CR) with 31,1 μL of 1 mg/mL type I collagen in 0.1 M acetic acid (Collagen type I from calf skin, acid soluble, Sigma), and neutralized with 1 M KOH. Then a cell pellet (2 × 106 cells), resuspended in 36 μL of α-MEM was added. Subsequently, 120 μL of Tissucol solution in aprotinine (fibrinogen 70–110 mg/mL, aprotinine 3000 KIU/mL), and 120 μL of thrombin solution (4 IU/mL) in CaCl2 (40 μmol/mL, Tissucol^® ^Kit, Baxter) were mixed and added to each well and a content of each well was thoroughly mixed. The gel was formed at 37°C for 15 min. Subsequently, the culture medium was added and the scaffold was placed in an incubator with a humidified atmosphere, 5% CO2 at 37°C and implanted on the same day.

### Surgical procedures

Surgeries were performed under general anaesthesia. Before anaesthesia, enrofloxacin (BAYTRIL 2.5% inj. ad us. vet., Bayer) was administered intravenously at the dose of 5 mg/kg. Induction was achieved by intramuscular administration of midazolam (1.00 mg/kg (DORMICUM inj., Roche) + fentanyl (0.02 mg/kg (FENTANYL, Janssen) and medetomidine at the dose of 200 μg/kg (DOMITOR inj. a.u.v., Pfizer). Total inhalation anaesthesia was then maintained by a mixture of oxygen, nitrous oxide (2 : 3) and isoflurane (FORANE, Abbott Laboratoires) using a non re-breathing system (Bain). The heart rate, respiratory rate, invasive blood pressure, end-tidal partial pressure of carbon dioxide and saturation of haemoglobin by oxygen was monitored (DATEX Cardiocap II). As this combination of drugs causes a strong respiratory depression, all animals were connected and controlled by ventilation device.

Rabbits were placed in dorsal recumbency and the surgical site was routinely prepared for the aseptic procedure on both knees. Lateral arthrotomy of the stifle joint was performed by parapatellar incision. After visual localization of the growth plate, the battery-powered drill (Colibri system, SYNTHES, USA) was used to create a defect in the lateral part of the distal femoral physis in order to cause damage exceeding 9% of the growth plate area [5,13,14]. Therefore, a 3.5 mm drill bit (ACUFEX – MosaicPlasty Precision, Smith&Nephew, USA) was used to bore a canal 12 mm deep from the lateral surface of the lateral condyle dorsolaterally above the insertion of m. extensor digitorum longus. The canal was drilled in the dorsomedial direction in order to cause damage of the lateral part of the distal femoral physis including the adjacent parts of epiphysis and metaphysis. The external part of the canal of the iatrogenic defect of the distal physis of the right femur was closed only with a cylinder made from beta-tricalcium phosphate (ChronOS, SYNTHES) 3.5 mm thick and 2 mm long, that was cut out from a pre-formed ChronOS block using a 3.5 mm tubular chisel (ACUFEX – MosaicPlasty Precision, Smith&Nephew, USA). This bioceramic cylinder was stained with methylene blue (ModR metylenovA, ind., 100 g, FISHER SCIENTIFIC) for easier orientation during the following arthrotomy with the bone bridge excision and therapeutic transplantation of MSCs.

The stifle joint was lavaged with Ringer Lactat solution (Ringer Lactat I.V.Inf., Braun Medical AG). The joint capsule was closed with an interrupted suture (polypropylene, Prolene 4/0, Ethicon). The subcutaneous layer was closed with a continuous suture using 2/0 polyglactin 910 (Vicryl, Ethicon). The skin was closed with a simple interrupted suture using 2/0 polyglactin 910 (Vicryl, Ethicon).

This iatrogenically created defect of the distal physis of the right femur in animals of Groups A and B then served as the site of transplantation of the gel scaffold with autogenous mesenchymal stem cells (Group A) or allogeneic MSCs (Group B). The defect of the growth cartilage was created using the same method in the left femoral bone and was left in all the animals in groups A and B without transplantation of MSCs and served in both groups as control.

Three weeks after causing the damage we performed arthrotomy of the right knee joint using the same method in animals of both groups A and B. The bioceramic cylinder stained with methylene blue and the bone bridge formed at the site of the original physeal defect were bored off by a drill with a 3.5 mm diameter (SYNTHES). Before transplantation of the scaffold with MSCs, the canal was dilated using a 3.5 mm dilator (ACUFEX – MosaicPlasty Precision, Smith&Nephew, USA). A mixture of the scaffold and MSCs (autogenous in Group A; allogeneic in Group B, respectively) was prepared in wells of a microtitration plate (TPT), from where the implant (in the form of a cylinder 3.5 mm thick and 10 mm long) was taken by the drill guide (ACUFEX – MosaicPlasty Precision, Smith&Nephew, USA) and carefully inserted using a delivery tamp (ACUFEX – MosaicPlasty Precision, Smith&Nephew, USA) into the defect drilled in the lateral right femoral condyle. In order to fix the transplant in its position, the canal was closed (on the lateral surface of the lateral condyle of the femur) with a cylinder made from beta-tricalcium phosphate (ChronOS, SYNTHES) 3.5 mm thick and 2 mm long, that was cut out from a pre-formed ChronOS block using a 3.5 mm tubular chisel (ACUFEX – MosaicPlasty Precision, Smith&Nephew, USA).

Antagonization of all three anaesthetic components was performed using a combination of naloxon (0.03 mg/kg) (INTRENON inj., Leciva a.s.) + flumazenil (0.1 mg/kg) (ANEXATE, Hoffmann-La Roche Ltd.) + atipamezol (1.0 mg/kg) (ANTISEDAN inj. ad us. vet., Pfizer Animal Health) administered intramuscularly after the surgery. Analgesia in the post-operative period was achieved by administration of carprofen (RIMADYL inj. ad us. vet., Pfizer Animal Health) at the dose of 2 mg/kg/day for three days after the surgery. The animals were allowed to walk freely and bear weight as tolerated following recovery from surgery. The animals were fed, handled and housed according to the principles of welfare during the whole study period. At the end of the experiment (4 months after the first surgery), all animals were euthanized lege artis. Firstly, they were put under general anaesthesia using intravenous thiopental at the dose of 20 mg/kg; then they were given intravenous T 61 inj. ad us. vet. (Hoechst Roussel Vet.) at the dose of 1 ml pro toto.

The length and angular (valgus) deformity of the operated bone were measured from radiographs in the craniocaudal (CC) projection. The quality of graft incorporation was evaluated histologically. The presence of transplanted cells in the physis was detected by immunofluorescence. All procedures were conducted with the consent of the Ethical Committee (No. 46613/2003-1020).

### Bone length discrepancy and femoral valgus deformity measurements

Each rabbit was subjected to radiological examination on the day of the first surgery (bone marrow blood harvesting), and after euthanasia. Bone length discrepancy and valgus deformity were measured from radiographs. Length measurement of the right femur (with the physeal defect and transplanted MSCs) and the left femur (with the physeal defect without transplanted MSCs) was done from radiographs of the femur in the craniocaudal (CC) projection. Actual length of the femur and the angle of valgus deformity of the distal femur were measured. The measurements were performed separately by three independent observers. The measured values were averaged to calculate the arithmetic mean.

### Magnetic resonance imaging

We used magnetic resonance imaging to in vivo detection of transplanted MSCs in the physeal defect. The rabbits were subjected to MRI examination three weeks after the surgery and on the day of euthanasia – they were examined by the technique of T1 weighed images and by the sequence modified to highlight the hyposignal of MSCs labelled with iron oxide (detection of paramagnetic iron oxide nanoparticles, Resovist). A three-week interval between the transplantation and the first MRI examination was allowed to eliminate possible formation of artifacts caused by postoperative haematoma [16].

### Histological findings

The defect healing was examined histologically using haematoxiline and eosin staining. Following the excision of femurs of the euthanized rabbits, femoral distal epiphyses were placed and stabilized in a 10% solution of formalin. They were then decalcified and gradually dehydrated in solutions with an increasing concentration of alcohol to enable them take to paraffin. Ultrathin paraffin sections of the distal femur 0.1 mm in thickness were stained with haematoxiline and eosin (HE) and subjected to microscopy, histochemical analysis – collagen – 2 immunostaining (Picture 4) and PAS reaction were provided. The defect site in the growth plate of the femur was examined histologically. The presence of the hyaline cartilage in the original defect was observed, as well as possible histological signs of transplant rejection (lymphocyte infiltrate, cartilage separation from the surrounding tissue, ligament degeneration). The ferrous stain Resovist incorporated in the MSC cytoplasma allowed the processing of one of the histological sections for Pearls reaction (staining with Berlin blue) and thus to verify the origin of chondrocytes from the transplanted MSCs.

These examinations should prove whether the chondrocytes present in the defect originated from the implanted colony of MSCs or not on the basis of immunofluorescence detection of the CM-DiI stain incorporated into the cell wall.

### Statistical evaluation

Means and standard deviations were calculated for the length and valgus deformity of the right femur (with the physeal defect and transplanted MSCs) and the left femur (with the physeal defect without transplanted MSCs) as well as for differences in length and angular deformities before MSCs transplantation and after euthanasia. The values were statistically analyzed using Wilcoxon matched-pairs test; STATISTICA (data analysis software system), version 7.1 (StatSoft, Inc. 2005).

## Results

No animal suffered perioperative complications or premature death. During the cultivation of MSCs, two experimental animals had to be transferred from Group A to Group B, as their autogenous graft did not reach the number of cells needed for a successful transplantation. Four months after the transplantation, the right femurs of animals of Group A (with the excised bone bridge and transplanted autogenous MSCs) were longer on average by 0.50 ± 0.04 cm (p = 0.018) than their left (control) femurs (without transplanted MSCs). 4 months after the transplantation the right femurs of rabbits of Group B (with the excised bone bridge and transplanted allogeneic MSCs) were longer on average by 0.43 ± 0.01 cm (p = 0.028) than their left (control) femurs (without transplanted MSCs).

4 months after the therapeutic transplantation of MSCs was the valgus deformity of the distal part of the right femur in the animals of Group A (with the excised bone bridge and transplanted autogenous MSCs significantly lower (by 4.45 ± 1.86°) than valgus deformity of the distal segment of their left (control) femur (without transplanted MSCs) (p = 0.028).

Likewise, valgus deformity of the distal part of the right femur in the animals of Group B (with the excised bone bridge and transplanted MSCs) was 4 months after the therapeutic transplantation of MSCs significantly lower (by 3.66 ± 0.95°) than valgus deformity of the distal segment of their left (control) femur (without transplanted MSCs) (p = 0.001).

However, no statistically significant difference was found in the length (i.e., the lengthwise growth) of the right femoral bone (p = 0.495) and its valgus deformity (p = 0.1597) between rabbits with transplanted autogenous MSCs (Group A) and rabbits with transplanted allogeneic MSCs (Group B).

**Figure 1:**
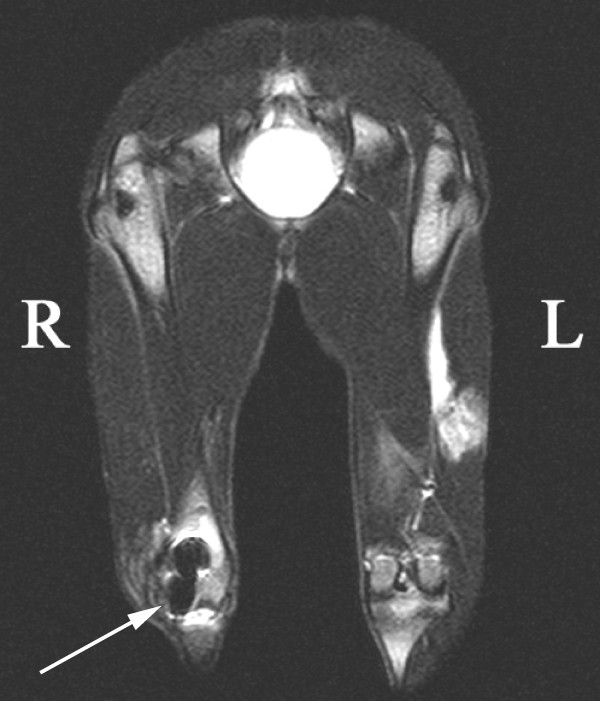
**Resovist contrast agent incorporated into transplanted MSCs**. The arrow shows an artifact created from paramagnetic iron nano-particles with Resovist contrast agent incorporated into transplanted MSCs (rabbit A5).

MRI examination proved the presence of paramagnetic nano-particles of iron oxide at the site of transplantation of labelled MSCs in the lateral section of the right femoral condyle in 100% of cases 3 weeks after the transplantation as well as 4 months after the transplantation (Fig. 1).

**Figure 2:**
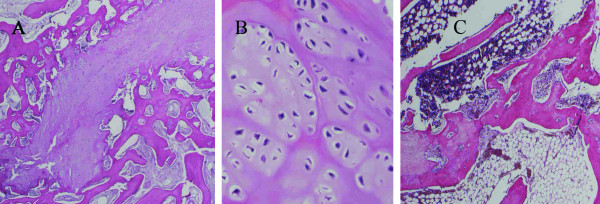
**Histological examination of the distal femoral physis in rabbits after autogenous MSC transplantation (HE stain)**. A – after autogenous MSC transplantation into the femoral defect (magnification × 40 – rabbit A10). B – after allogeneic MSC transplantation into the femoral defect (magnification × 100 – rabbit B3). C – left femur physeal defect without MSC transplantation – bone bridge was formed (magnification × 20 – rabbit B6 – left femur).

**Figure 3:**
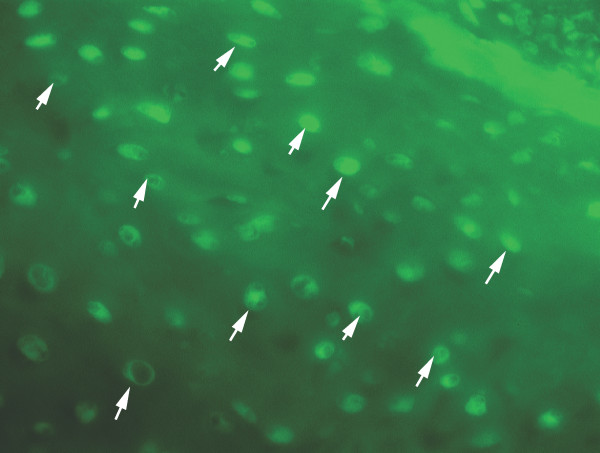
Immunofluorescence stain DiI in chondrocyte membranes (arrows) differentiated from implanted allogeneic MSCs (rabbit A5× 400).

**Figure 4:**
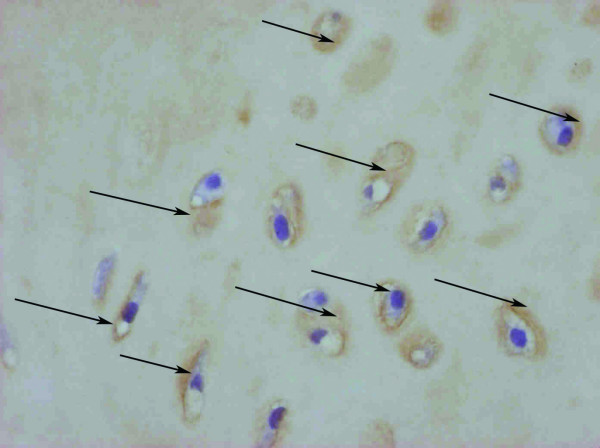
Histochemical analysis – collagen – 2 immunostaining (arrows), positive result (rabbit A2 × 100).

The presence of newly formed hyaline cartilage was confirmed by histological examination at the site of the original defect of the growth plate of the right distal femur in all the animals of both groups A and B (Fig. 2). No histological signs of allogeneic transplant rejection were found in the microscopic sections of the distal physis of the right femur (experimental animals of Group B with implanted allogeneic MSCs). A bone bridge was histologically demonstrated in all cases in the distal physis of the left femur with an iatrogenic growth cartilage defect without transplanted MSCs (Fig. 2). Pearls reaction in the histological preparation of the distal physis of the right femur of all the animals of both groups A and B was positive. The presence of particles of the lipophilic stain DiI was confirmed by immunofluorescence in all the histological samples of the distal growth zones of the right femoral bones with the exception of one case in Group A (MSC autotransplantation) and one case in Group B (MSC allotransplantation) (Fig. 3). Histochemical analysis – collagen – 2 immunostaining (Fig 4) and PAS reaction was positive in all the histological samples of the distal growth zones of the right femoral bones in Group A and B.

## Discussion

In our experimental study, we tried to find whether there is difference in the results of a bone bridge treatment by its excision and transplantation of mesenchymal stem cells in dependence on the type of the cellular implant used (autogenous vs. allogeneic MSCs). Thus, the fundamental question is whether we can expect the same clinical results from autogenous and allogeneic transplantation of MSCs.

It is appropriate to investigate both methods for future clinical practice. Allografts may be potentially used in both preventive and therapeutic transplantation into the damaged growth plate. The bone bridge formation may be detected approximately 4 weeks after injury by radiological or CT examination or by magnetic resonance [17,18]. If diagnosing a physeal closure by a bone bridge, it would then be possible to use the cultivated autogenous MSCs collected from the patient when the trauma originated. If the bone bridge, representing a risk of growth disorder of the injured bone (9% of the physeal area) [5,13,14], did not form, it would be possible to keep the unused cells in the tissue bank as a potential MSC allotransplant.

The use of progenitors of chondrogenic differentiation, i.e. stem cells of mesenchymal origin, started to present itself immediately after the first promising experiments with transplantation of autogenous/allogeneic chondrocytes [13,14]. Apart from the bone marrow the source of MSCs may also be the synovial fluid, periosteum, adipose tissue and partly also muscular tissue [8,19]. Their collection should not be burdensome for the potential donor or the patient him/herself. To keep MSC allotransplants successfully in tissue and cell banks and to use them without further delay in case of need in an acute injury of the growth plate if the future shows that it is possible in experiments and subsequently in clinical practice, the method of treating physeal plate traumas by preventive transplantation of MSCs would appear perspective [6]. A suitable scaffold that would provide the cells not only with sufficient space and mechanical support for growth but also with an adequate supply of nutrients could be prepared e.g. on the basis of IPN (Interpenetrating Polymer Network). Rightly timed cascade of other factors that may influence *in vivo *the intercellular matrix is needed apart from the initiation of cell differentiation by supplementing some recombinant factors. In the abovementioned cytology laboratories, the differentiation scheme with the use of TGF-β1 [15] was repeatedly effective in experiments. In general, TGF-β1 and other members of TGF superfamily (BMP2, BMP4) are used for chondrogenic differentiation of MSC cells and TGF is the most „traditional”. Most strikingly BMP2 is also used in several studies to promote chondrogenesis although it preferentially stimulates osteogenesis. To our knowledge more than growth factor used culture conditions are more imortant for targeted differentiation. As chondrogenesis and osteogenesis display similar initiation processes (nodule formation and collagen deposition) related molecules sucg as TGF and BMP can substitute each other. Our in vitro observations and published articles by other authors indicate that osteogenesis takes place mainly in confluent-like 2D culture while chondrogenesis requires high cell density 3D culture and reduction of cell adhesion to rigid surface. Other supplements in differentiation media (dexamethasone and ascorbic acid) are stimulatory for collagen biosynthesis without any direct effect to differentiation signalling.

The surgical procedure and method of creating the iatrogenic defect in the growth cartilage was chosen in this study on the basis of good experience with methodology used in our previous experiments [6,7,13,14] and in similar studies of other authors [9,20].

These rabbits attain full femoral maturity at approximately 4 – 6 months of age [9,20,21], our observation period (5 week by the operation, 4 months observation period) consists with this reality. The iatrogenic damage of the growth cartilage and in order to make the biggish defect (7 – 9% of growth plate area) described Janarv [5].

Radiological examination is easily accessible and allows determining with a relatively high accuracy both the length of the x-rayed bone and its possible angular deformity. The method of measuring the femoral bone length in the rabbit was based on Janarv's original study [5], whereas the measuring of the extent of valgus deformity had been consulted with specialists on descriptive geometry previously for the needs of earlier studies of the authors [6,7].

The detection of paramagnetic iron nanoparticles of the phagocyted MSCs in the preparatory phase *in vitro *using MRI is very favourable. In live animals it allows us to detect, i.e. before the end of the experiment, their presence at the site of transplantation, or to find their extinction or travel from the destination site, etc.

Histological examination of microsections stained with haematoxiline and eosin (HE) gives a reliable answer to the character and quality of the cartilage in the iatrogenically created defects. The results of our examinations were fully in accordance with the established hypothesis and also corresponded with the results of several similar studies [9,20]. The presence of bright blue granules in the chondrocyte cytoplasma (Resovist combined with Berlin blue – Pearls reaction) and immunofluorescence stain CM-DiI [9] reliably determined the origin of the cells from the MSC transplant used by out team.

The results of our work indicate that the transplantation of mesenchymal stem cells into the physis at the site of the excised bone bridge may prevent the shortening of the affected bone and the occurrence of angular deformities. At the same time, the study confirmed in an animal model that there is no qualitative difference (hyaline cartilage) in the character of the newly formed cartilaginous tissue in the use of autogenous vs. allogeneic MSCs. In clinical use, both methods of MSC transplantation (auto- and allotransplantation) may have their justification, in dependence on the given clinical case and circumstances of treatment.

## Conclusion

Allogeneic as well as autogenous transplantations of MSCs into the growth zone defect after the bone bridge excision prevented limitation of the bone growth lengthwise and prevented the development of its angular deformity. Concurrently, no difference was found between the results of allogeneic and autogenous transplantation of MSCs. Both from the viewpoint of the lengthwise bone growth and its potential angular deformity. From the viewpoint of the quality of the newly formed cartilaginous tissue at the site of the original physeal defect. Following autogenous as well as allogeneic transplantations of MSC, the presence of hyaline cartilage was histologically confirmed at the site of the treated growth plate defect. In the case of allotransplants, no histological signs of their rejection were found. At the same time it was demonstrated in vivo and in vitro that the chondrocytes newly formed at the site of the physeal defect originated from the transplanted mesenchymal stem cells.

## Authors' contributions

LP, PG and AN carried out the surgical procedures, conceived of the study and participated equally in the design of this experimental work, performed the statistical analysis, and drafted the manuscript. HK, RS, LU and JL participated in surgical procedures, carried out perioperative care of experimental animals, and were responsible for sampling of tissues submitted to histological evaluation. JK and JH carried out isolation, culturing and labelling of mesenchymal stem cells. EF and EA were responsible for scaffold preparation. PK carried out diagnostic imaging examinations. LK carried out histological examinations and interpreted histological findings. All authors read and approved the final manuscript.
